# UV-B Filter Octylmethoxycinnamate Is a Modulator of the Serotonin and Histamine Receptors in Human Umbilical Arteries

**DOI:** 10.3390/biomedicines10051054

**Published:** 2022-05-03

**Authors:** Margarida Lorigo, Carla Quintaneiro, Luiza Breitenfeld, Elisa Cairrao

**Affiliations:** 1 CICS-UBI, Health Sciences Research Centre, University of Beira Interior, 6200-506 Covilhã, Portugal; margarida.lorigo@gmail.com (M.L.); luiza@fcsaude.ubi.pt (L.B.); 2 FCS-UBI, Faculty of Health Sciences, University of Beira Interior, 6200-506 Covilhã, Portugal; 3 Department of Biology & CESAM (Centre for Environmental and Marine Studies), University of Aveiro, 3810-193 Aveiro, Portugal; cquintaneiro@ua.pt

**Keywords:** personal care product, endocrine-disrupting chemical, pregnant women, preeclampsia, gestational hypertension

## Abstract

Every day, people use personal care products containing UV filters. Although their use initially showed a protective role, toxicity is a concern for human health as several UV filters are endocrine-disrupting chemicals (EDCs). Exposure to EDCs may induce cardiovascular diseases and can affect the health of sensitive people, such as pregnant women. Currently, the world’s most widely used UV-B filter is octylmethoxycinnamate (OMC), an EDC. However, the disruptive effects on pregnant women are little known. The present work proposed to understand how long-term exposure to OMC affects vascular homeostasis. Endothelium-denuded human umbilical artery (HUA) rings were incubated in an organ bath system. Long-term effects of exposure to OMC (0.001–50 μmol/L) were evaluated on the contractile responses of HUA to the application of the contractile agents, serotonin (5-HT) and histamine (Hist). To investigate in more detail the vascular mode of action of OMC, through which it impairs the vascular homeostasis of HUA, the activity and expression of different 5-HT and Hist-receptors involved in contractility processes were studied. Our findings pointed out an increase in the reactivity of HUA to 5-HT and Hist due to OMC exposure. These alterations in reactivity may be precursors of preeclampsia development and/or gestational hypertension.

## 1. Introduction

UV-B filters are designed to protect against the photobiological effects of radiation [1,2], such as sunburn, pigmentation problems, immunosuppression, and damage to DNA and other cellular structures [3,4]. However, the most harmful effect of carcinogenesis seems not to be diminished [5,6].

Octylmethoxycinnamate (OMC) is one of the world’s most widely used UV-B filters. However, OMC is an endocrine-disrupting chemical (EDC) and its effects on human health and the environment are currently the subject of great controversy [1,7,8,9]. Recent studies have shown that OMC can cause severe damage to coral reefs, which is why it was taken off the market in Hawaii in 2018 [9,10]. On the other hand, concerning the disrupting effects of OMC in humans (recently reviewed [7]), in vitro studies pointed to estrogenic, anti-progestogenic, and anti-thyroid activities; while androgenic or anti-androgenic activities were not proven in human cells [11,12].

Thus, although the Food and Drug Administration (FDA) has approved the use of OMC in specific concentrations, its effects on humans still do not reach consensus within the scientific community, and if OMC is banned, 90% of cosmetic products will be taken off the market and this will have a major economic impact [8]. Therefore, OMC, acting as an EDC, has the capacity to interfere with endogenous hormones [13], which is a concern for human health, particularly in populations more sensitive to endocrine disruption, like pregnant women and developing foetuses [14,15,16]. In this sense, the human umbilical artery (HUA) is an excellent model to study the disrupting effects of EDCs on the vascular system [17,18]. The umbilical cord is easy to obtain after birth, is often discarded, and has been widely used as a model in cardiovascular studies [19] and tissue engineering [20]. In addition, the HUA can be easily isolated from the umbilical cord and is an excellent source of vascular smooth muscle cells (SMC), allowing contractility studies at arterial and cellular levels [18]. These studies will allow us to better understand the correlation between human exposure to EDCs and vascular diseases, such as gestational hypertension or preeclampsia (PE).

Recently, we reported that OMC acts as a rapid vasodilator of human umbilical arteries [21]. Similar to estrogens, our results suggested that the endothelium-independent vasorelaxation induced by OMC appears to be due to the inhibition of L-type voltage-operated Ca^2+^ channels (L-Type VOCC) and activation of soluble guanylyl cyclase (sGC), increasing intracellular cGMP levels [21]. However, in the long-term, this effect appears to be reversed, inducing vasoconstriction of the HUA by mechanisms opposite to those observed previously, thus indicating that OMC may be an inductor of hypertensive disorders of pregnancy (HDP) by impairing the vascular system of pregnant women [22].

On the other hand, the HUA is mainly regulated by local mediators (serotonin and histamine) [17,23]. The effects of these mediators are induced by membrane receptors (such as G protein-coupled receptors, GPCR), which, when altered, compromise maternofoetal health. Namely, elevated levels of serotonin and histamine increase HUA reactivity/sensitivity to these vasoactive agents, increasing vascular resistance and inducing PE and gestational hypertension [24,25,26,27,28].

Therefore, since EDCs can activate membrane receptors and alter signal transduction [29], the novelty of our work is to understand how long-term exposure to OMC affects vascular homeostasis by modulating serotonin and histamine receptors, and ultimately inducing HDP. To elucidate this, the effects of long-term exposure to OMC were evaluated on contractile responses to serotonin and histamine of endothelium-denuded HUA-rings. Since OMC altered the vascular homeostasis of arteries, their vascular mode of action (MOA) was explored in more detail by analysing the activity of the different 5-HT and Hist receptors involved in the contractility response. Furthermore, the HUA was also used to perform cultures of vascular SMC, which were used to analyse the expression of the different 5-HT and Hist receptors through real-time PCR.

## 2. Materials and Methods

The present work was performed according to the Declaration of Helsinki principles and approved by the Ethics Committees for health from local hospitals (CHUCB, No.33/2018, 18 July 2018; and ULS-Guarda, No.02324/2019, 27 February 2019).

### 2.1. Sample Collection

The human umbilical cord was chosen as a vascular model to study the vascular implications of exposure to OMC in pregnancy. According to our previous studies, this is the appropriate model for an endocrine disruption study [17,18,30] since the HUA is readily isolated and an excellent source of SMC, allowing different studies at the vascular level.

Sixty-five pieces of the human umbilical cord of normal full-term pregnancies were collected in the CHUCB and ULS-Guarda. Sample collection was performed from healthy pregnant women after signing an informed consent form. This investigation did not include pregnant women with significant medical disorders. Umbilical cord samples were collected immediately after vaginal delivery and stored at 4 °C (during 4–24 h) in a sterile solution (PSS, physiological saline solution) with the composition (mmol/L): 0.50 EDTA, 5 KCl, 10 HEPES, 2 MgCl_2_, 10 NaHCO_3_, 0.5 KH_2_PO_4_, 0.5 NaH_2_PO_4_, 10 Glucose, 110 NaCl, and 0.16 CaCl_2_ (pH = 7.4). To avoid tissue contamination and degradation, some antibiotics and antiproteases were added to the sterile solution that contained the umbilical cord. The antibiotics were 5 U/mL penicillin, 5 μg/mL streptomycin, and 12.5 ng/mL amphotericin B, and the antiproteases used were 0.45 mg/L leupeptin, 26 mg/L benzamidine, and 10 mg/L trypsin inhibitor.

### 2.2. Preparation of HUA-Rings

Preparation of the human arteries was performed as described previously [22]. Briefly, the HUA were dissected from Wharton’s jelly and cut into small rings (3–5 mm in length). Then, the vascular endothelium was removed to evaluate the effects of OMC at the smooth muscle level only. The long-term effects of OMC on HUA contractility were assessed over a pre-incubation period (24 h) with OMC (0, 1, 10, and 50 μmol/L) before any experiment, as per Lorigo, et al. (2021) [22]. Our research group [22,30] demonstrated that 24 h of pre-incubation with an EDC is enough time to change the genomic profile involved in the HUA contractility response.

### 2.3. Vascular Reactivity Experiments

According to our previous works, the tension of the HUAs was measured [21,30]. Following the equilibration period, the viability of the rings was tested by pre-contracting them with serotonin at their supramaximal concentration (5-HT, 1 μmol/L). Rings with a maximum contraction less than 10 mN were not included.

Firstly, to directly evaluate the effect of OMC on the basal tension of the HUAs, pre-incubated rings were exposed to cumulative concentrations of OMC (0.001–50 μmol/L) [21,31]. Then, the rings were contracted with serotonin (5-HT, 1 μmol/L) to test their contractile response.

A second experiment was done to evaluate the effects of OMC on contraction after the artery was contracted with two contractile agents, such as serotonin (5-HT) and histamine (Hist). Briefly, pre-incubated rings were contracted using 5-HT (1 μmol/L) or Hist (10 μmol/L) until achieving a plateau phase. After this phase, rings were exposed to a cumulative dose of OMC (0.001–50 μmol/L), and the tension was determined. A control was performed using a solvent (ethanol) at the same percentage used in the dissolution of OMC.

Further studies were performed to identify the long-term effects of OMC on 5-HT and Hist receptors. The role of receptors on the regulation of vascular contractility was determined using the supramaximal concentrations of their specific agonists and antagonists. The following procedures were performed:

1. The HUA rings were contracted using an agonist of 5-HT_2A_ receptors, alpha-methyl-5-hydroxytryptamine (AMHT; 1 µmol/L), and after reaching a stable contraction, 1 µmol/L of AS19, an agonist of 5-HT_7_ receptors, was added to each ring, and the % of relaxation was measured.
2. The HUA rings were contracted using the 5-HT_1B_ and 5-HT_1D_ receptor agonist, L-694247 (L69; 1 µmol/L). In responsive HUA-rings, after reaching a stable contraction, 1 µmol/L of AS19, an agonist of 5-HT_7_ receptors, was added to each ring and the % of relaxation was measured.
3. The HUA rings were contracted using an agonist of H_1_ receptors, betahistine (BHI, 1000 µmol/L), and after reaching a stable contraction (~10 min), 100 µmol/L of dimaprit, an agonist of H_2_ receptors, was added to each ring and the % of relaxation was measured.
4. The HUA rings were exposed to an antagonist of the H_2_ receptor, cimetidine (10 µmol/L), and after ~15 min, 10 µmol/L of Hist, an unspecific agonist of Hist receptors, was added to each ring and the % contraction was measured.

OMC photodegradation was avoided by the vascular reactivity experiments being carried out with no UV-light exposure.

### 2.4. Smooth Muscle Cells Dissociation and Culture

Cultures of SMC were performed according to Lorigo, et al. (2019) following the explant method of the HUA [21]. The SMC cultures were kept in an incubator with the following parameters: 37 °C, in an atmosphere of 95% O_2_ and 5% CO_2_.

The composition of the culture medium for cell growth was DMEM-F12 supplemented with BSA (bovine serum albumin; 0.5%), FBS (heat-inactivated foetal bovine serum; 5%), EGF (epidermal growth factor; 5 μg/mL), FGF (fibroblast growth factor; 0.05 µg/mL), heparin (2 μg/mL), insulin (5 μg/mL), and a mixture of antibiotics (penicillin, 5 U/mL; streptomycin, 5 μg/mL; and amphotericin B, 1.25 µg/mL).

When a confluent culture was reached, cells were trypsinized using a commercial trypsin-EDTA solution (0.025%), and subcultures of these cells were used until passage four. Cells from different passages were used to extract the total RNA and determine gene expression. Before each experiment, confluent HUASMCs were placed in a culture medium without FBS for 24 h and cultured as above, allowing the SMC to express the contractile phenotype.

### 2.5. Real-Time Quantitative Polymerase Chain Reaction (qPCR)

Confluent HUASMCs were subjected to OMC at the different concentrations (0.001–50 μmol/L) for 24 h. According to the manufacturer’s recommendations, the total RNA was extracted from cells using the Tri reagent™ (Ambion, Paisley, UK)). The RNA quantity was determined by spectrophotometry at 260 nm and 280 nm (Pharmacia Biotech, Ultrospec 3000, Cambridge, UK). Furthermore, the RNA quality was assessed by electrophoresis with a 1% agarose gel. According to the manufacturer’s (NZYTech, Lisbon, Portugal) recommendations, cDNA synthesis was achieved using 1 µg of the total RNA. Gene-specific primers (Table 1) were chosen to determine the gene expression of the serotonin (5-HT_2A_) and histamine (H_1_) receptors by qPCR using SYBR Green qPCR Master Mix (NZYTech, Lisbon, Portugal). Gene expression levels were normalized using human β-actin as the housekeeping gene. Samples were amplified with an iQ5 system (Bio-Rad, Hercules, CA, USA) programmed as follows: denaturation at 95 °C for 5 min, followed by 30 cycles (10 s each) at 95 °C, annealing for 30 s at 60 °C and extension for 10 s at 72 °C. The amplified qPCR fragments were analysed by melting curves: reactions were heated from 55 °C to 95 °C with 10 s holds at each temperature (0.05 °C/s). The mathematical model proposed by Pfaffl (2001) [32] was used to calculate fold changes in mRNA expression using the formula 2^−ΔΔCt^.

### 2.6. Drugs and Chemicals

All drugs and chemicals used in this work were purchased from Sigma-Aldrich Química (Sintra, Portugal), except dimaprit and L69, which were purchased from Tocris Bioscience (Bristol, UK) and AS19 from Biogen Cientifica (Madrid, Spain). The ethanol control and the OMC solutions were prepared daily by dilution with appropriate solutions (Krebs’ solution or FBS-free culture medium) according to the experiment. To avoid cytotoxicity, the final concentration of ethanol in the experiments never exceeded 0.05%.

### 2.7. Statistical Analysis

Measurements of isometric tensions were expressed in millinewtons (mN) of force elicited by the HUA in the presence of the drugs. Relaxation data was analysed by the percentage reduction in the maximal contraction induced by 5-HT and His. The results were expressed as the mean ± S.E.M. of the number (*n*) of rings used.

SigmaStat v.3.5 (2006) software (Systat Software, San Jose, CA, USA) was used to perform the statistical analysis. All data were checked for normality and the homoscedasticity criteria using Kolmogorov–Smirnov and Levene’s mean tests, respectively. Differences in basal tension and the % HUA relaxation were analysed by ANOVA followed by pairwise Dunnett’s post hoc tests. Moreover, in the cases where normality and/or homoscedasticity were not verified, a non-parametric Kruskal–Wallis test was performed.

In the analysis of the effect of OMC on the HUA contracted by serotonin, or the effect of OMC on the HUA tension by agonists/antagonists of serotonin and histamine, the two-way ANOVA followed by the Holm–Sidak post hoc test was applied. This procedure allowed us to compare the interaction between factors (pre-incubation with OMC and cumulative doses of OMC or the agonist/antagonist tension) and identify the significant differences. Whenever the set data did not follow a normal distribution, it was log_10_ transformed. For the gene expression results, the Student’s *t*-test was performed to detect differences in the mRNA expression between the control and treatment groups. Graphic design of the data was achieved using the software Origin 8.5.1. (OriginLab, Northampton, MA, USA). In all the statistical analysis, *p* < 0.05 was considered statistically significant.

## 3. Results

### 3.1. Direct Effects of OMC on Basal Tension of the Human Vasculature

**Figure 1:**
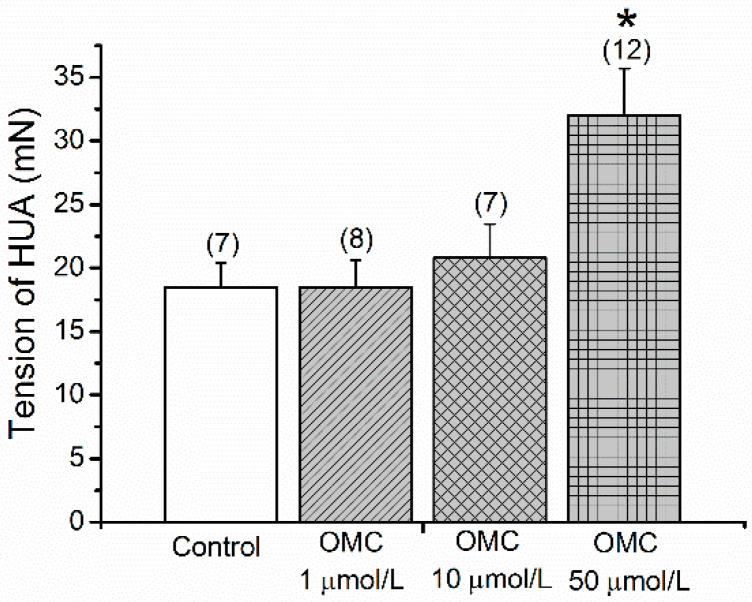
Tension induced by serotonin (5-HT, 1 µmol/L) after exposure to cumulative concentrations of octylmethoxycinnamate (OMC; 0.001–50 μmol/L) on the human umbilical artery (HUA)-rings incubated with OMC. Data are presented as mean (bars) ± standard error of mean (S.E.M.) of the number *n* within brackets. * Represents a significant difference against HUA control (*p* < 0.05, one-way ANOVA with Dunnett’s).

The basal tension of the HUA was not changed in the presence of the various concentrations of OMC (0.001–50 μmol/L) (Supplementary Table S1). Figure 1 presents the tension of the pre-incubated rings exposed to cumulative doses of OMC and contracted with 5-HT. The results showed that the rings pre-incubated with the highest concentration (50 μmol/L) presented a significantly higher tension than the control rings (*p* < 0.05, one-way ANOVA with Dunnett’s).

### 3.2. Long-Term Effects of OMC on Human Arteries Contracted with Serotonin

Stable contractions of pre-incubated rings contracted with 5-HT were obtained after 15 min for all incubated arteries (data not shown). Similar tensions for HUA-rings incubated with all OMC concentrations were obtained, compared with the HUA-rings from the control (*p* > 0.05, one-way ANOVA).

**Figure 2:**
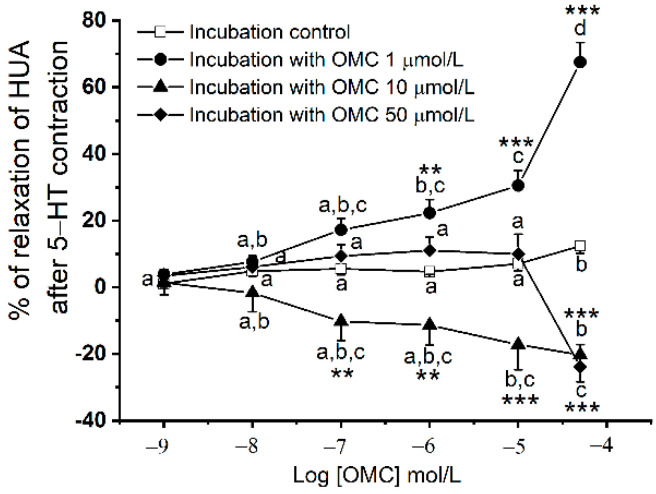
Percentage of relaxation of human umbilical artery (HUA)-rings incubated with octylmethoxycinnamate (OMC), contracted with serotonin (5-HT; 1 µmol/L) and exposed to cumulative concentrations of OMC (0.001–50 μmol/L, which is presented as Log [OMC]). Each point represents the mean and vertical lines represent the S.E.M. * Represents a significant difference when compared to rings from the HUA control: ** (*p* < 0.01) and *** (*p* < 0.001); and the different letters represent significant differences between OMC concentrations (*p* < 0.05); two-way ANOVA method followed by Holm–Sidak post hoc tests.

The long-term effects of exposure to OMC (by adding cumulative concentrations) on the 5-HT contracted HUA-rings are presented in Figure 2. The relaxation (%) induced by OMC was dependent on the pre-incubation concentration (Figure 2), as a significant interaction between pre-incubation with OMC and the different cumulative concentrations applied were observed (*p* < 0.001).

Regarding the control HUA-rings, the highest concentration of OMC (50 μmol/L) induced a significantly higher % of relaxation (*p* < 0.05). With respect to the HUA rings pre-incubated with the lower OMC concentration, there was a significantly higher % of relaxation for the concentrations of OMC, 1 μmol/L (*p* < 0.01), 10 μmol/L, and 50 μmol/L (*p* < 0.001). Concerning HUA pre-incubated with the intermediate concentration of OMC , a significant vasoconstriction (negative % of relaxation) was observed for the concentrations of OMC, 0.1 μmol/L, 1 μmol/L (*p* < 0.01), 10 μmol/L, and 50 μmol/L (*p* < 0.001). In the HUA pre-incubated with maximum concentration of OMC, the % OMC-induced relaxation was significantly lower for the highest concentration (50 μmol/L, *p* < 0.001). In the control groups, the solvent used (ethanol) did not have significant relaxant effects (*p* > 0.05, one-way ANOVA, see Supplementary Figure S1).

### 3.3. Long-Term Effects of OMC on Vascular Responses of Human Arteries to Agonists and Antagonists of Different 5-HT Receptors

**Figure 3:**
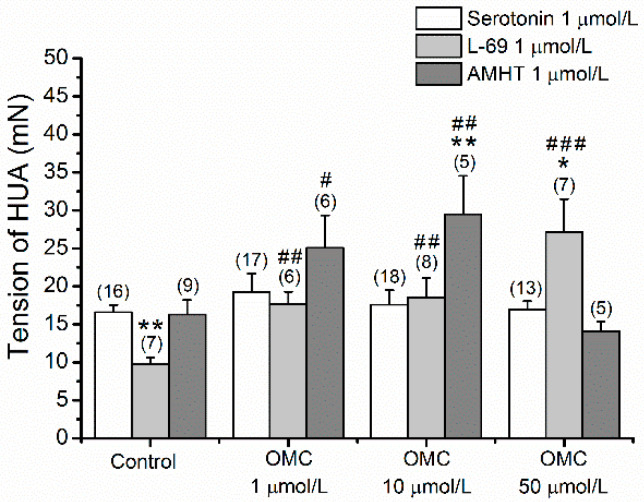
Tension of the human umbilical artery (HUA)-rings incubated with octylmethoxycinnamate (OMC) and contracted by serotonin (5-HT; 1 μmol/L), L-694247 (L69, 5-HT1_B/D_ agonist; 1 μmol/L), and by alpha-methyl-5-hydroxytryptamine (AMHT, 5-HT_2A_ agonist; 1 μmol/L). Each bar represents the mean, vertical lines represent the S.E.M., and the number within brackets is *n*. * Represents a significant difference when compared to the tension induced by 5-HT: ** (*p* < 0.01), and # represents a significant difference when compared to the tension of control group: ^#^ (*p* < 0.05), ^##^ (*p* < 0.01) and ^###^ (*p* < 0.001); two-way ANOVA method followed by Holm–Sidak post hoc tests.

Long-term effects of pre-incubation with OMC on 5-HT contracted HUAs pointed to the interference of OMC with serotonin receptors. In this sense, we analysed the involvement of 5-HT receptors (5-HT_2A_, 5-HT_1B_, 5-HT_1D_, and 5-HT_7_) in response to OMC exposure. The tensions observed in denuded HUA-rings pre-incubated with OMC and contracted using serotonin, AMHT (an agonist of the 5-HT_2A_), and L69 (an agonist of the 5-HT_1B_ and 5-HT_1D_ receptors) are presented in Figure 3. Statistical interaction between pre-incubation with OMC and the agonists of the 5-HT receptors analysed were verified (*p* < 0.001). The 5-HT_2A_ agonist, AMHT, induced a bigger contractility response in the HUA incubated with 1 μmol/L (2.507 ± 0.428 g) and 10 μmol/L (2.949 ± 0.507 g) (*p* < 0.05 and *p* < 0.01, respectively), than the control HUA(1.629 ± 0.191 g). Comparing the serotonin contraction effect with the agonist of 5-HT_2A,_ AMHT, the effect induced by AMHT was significantly higher (*p* < 0.01) in the HUA pre-incubated with OMC 10 μmol/L.

Regarding the HUA-rings contracted by L69, an agonist of 5-HT_1B_ and 5-HT_1D_ receptors, the results showed significantly higher tensions in the pre-incubated HUA-rings for all concentrations of OMC, than in the control group (0.974 ± 0.089 g), with 50 μmol/L presenting the highest tension (2.712 ± 0.437 g, *p* < 0.001). Concerning serotonin, the tension observed was significantly lower in the control group HUA-rings (*p* < 0.01) and considerably higher in the HUAs incubated with 50 μmol/L of OMC (*p* < 0.05).

L69 did not induce the same contractile effect in all HUA-rings. Supplementary Table S2 presents the number and tension of responsive and unresponsive HUA-rings to L69. The number of uncontracted HUA-rings is more pronounced for the pre-incubated arteries with 10 μmol/L (*n* =17) and 50 μmol/L of OMC (*n* = 26).

**Figure 4:**
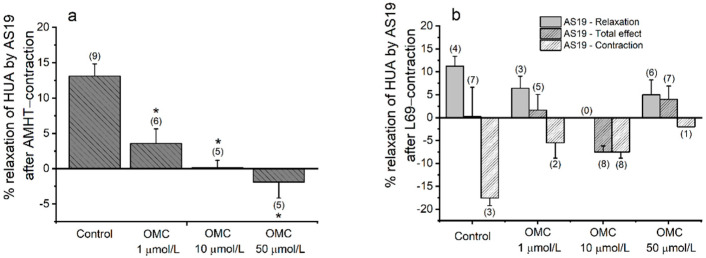
Percentage of relaxation of the human umbilical (HUA)-rings incubated with octylmethoxycinnamate (OMC), contracted by (**a**) alpha-methyl-5-hydroxytryptamine (AMHT, 5-HT_2A_ agonist; 1 μmol/L), and by (**b**) L-694247 (L69, 5-HT_1B/D_ agonist; 1 μmol/L) and relaxed by the agonist of the 5-HT_7_ receptor, AS19 (1 μmol/L). In (**b**), the dual effect of L69 is represented for three bars: relaxation (left), contraction (right), and the total effect (middle). Each bar represents the mean, vertical lines represent the S.E.M. and the number within brackets is *n*. * Represents a significant difference when compared to rings from the HUA control (*p* < 0.05).

The interference with the 5-HT_7_ receptor, which, when activated, induces relaxation, was evaluated using an agonist of the receptor AS19 (Figure 4). The % of relaxation induced by AS19 on HUA-rings contracted with the agonist of 5-HT_2A_ receptors (AMHT), was significantly lower in the pre-incubated HUA-rings (*p* < 0.05, one-way ANOVA with Dunnett’s) than the control HUA-rings (13.103 ± 1.728%, Figure 4a). On the HUA-rings contracted with the agonist of 5-HT_1B_ and 5-HT_1D_ receptors (L69), the response of the control group rings was not homogeneous (Figure 4b), half of the arteries constricted, and the other half relaxed, which led to a total percentage of relaxation close to zero. The same type of response was observed in HUA pre-incubated with the lowest concentration of OMC (1 μmol/L). However, rings incubated with 10 μmol/L of OMC presented only constriction (negative percentage of relaxation), and rings pre-incubated with 50 μmol/L showed a positive percentage of relaxation. The total effect of AS19 on L69-contracted HUA was similar for all OMC incubations compared with the HUA-rings from the control (*p* > 0.05, Kruskal–Wallis).

### 3.4. Long-Term Effects of OMC on Human Arteries Contracted with Histamine

**Figure 5:**
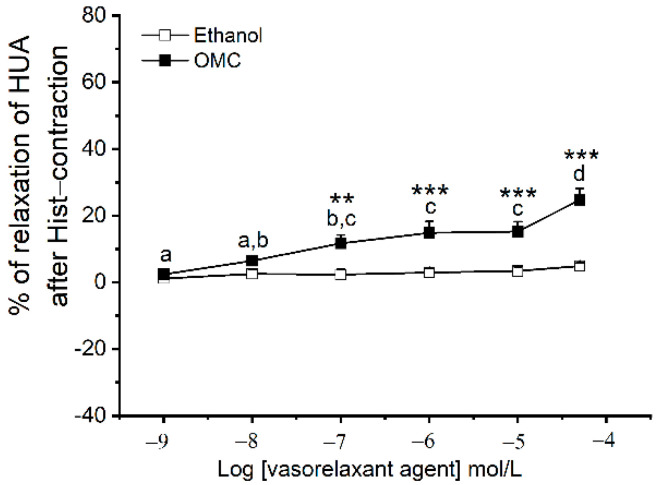
Percentage of relaxation of human umbilical artery (HUA)-rings incubated with octylmethoxycinnamate (OMC), contracted with histamine (Hist; 1 µmol/L) and exposed to cumulative concentrations of OMC (0.001–50 μmol/L). Each point represents the mean and vertical lines represent the S.E.M. * Represents a significant difference when compared with the respective solvent control: ** (*p* < 0.01) and *** (*p* < 0.001); and the different letters represent significant differences between OMC concentrations (*p* < 0.05); two-way ANOVA method followed by Holm–Sidak post-hoc tests.

Stable contractions of pre-incubated rings contracted with Hist were obtained after 15 min, only for HUA-rings from the control group (see Supplementary Figure S2). In these arteries, the maximum tension elicited by Hist was 1.239 ± 0.114 g (*n* = 8). Figure 5 presents the effects of exposure to cumulative concentrations of OMC on Hist-contracted HUA-rings from the control group. The maximum relaxation induced by OMC was 24.74  ±  3.40% (*n* = 12) and was observed at the highest tested concentration of OMC (50 μmol/L).

### 3.5. Long-Term Effects of OMC on Vascular Responses of Human Arteries to Agonists and Antagonists of Different Hist Receptors

**Figure 6:**
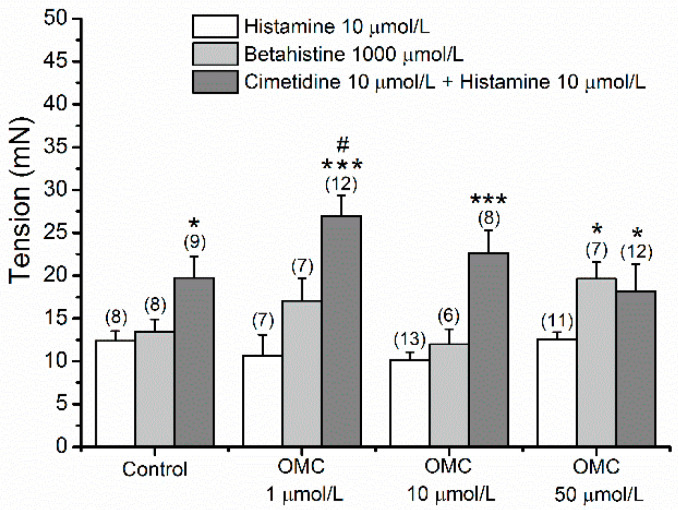
Tension (at time 15 min) of the human umbilical artery (HUA)-rings incubated with octylmethoxycinnamate (OMC) and contracted by histamine (Hist; unspecific agonist of Hist receptors; 10 μmol/L), betahistine (BHI; H_1_ agonist; 1000 μmol/L), and joint application of cimetidine (H_2_ antagonist; 10 μmol/L) and Hist (10 μmol/L). Each bar represents the mean, vertical lines represent the S.E.M. and the number within brackets represents *n*. * Represents a significant difference when compared to the tension induced by the Hist: * (*p* < 0.05) and *** (*p* < 0.001); # represents a significant difference when compared to the tension of control group: ^#^ (*p* < 0.05); two-way ANOVA method followed by Holm–Sidak post-hoc tests.

The long-term effects of pre-incubation with OMC on Hist-contracted human arteries clearly pointed to the interference of OMC on Hist receptors. In this sense, the involvement of H_1_ and H_2_ receptors in non-sustained contractions observed after OMC exposure was analysed. Figure 6 presents the tensions observed in incubated denuded HUA-rings contracted using histamine, an agonist of H_1_ (betahistine), and the joint application of an antagonist of H_2_ (cimetidine) and histamine. Tension recordings were presented at the time = 15 min to compare and analyse the involvement of OMC with the different Hist receptors (Figure 6).

No significant interaction between OMC pre-incubation and the agonists/antagonists of Hist receptors was observed (*p* = 0.05). However, the tension produced by the joint application of cimetidine + Hist in HUAs incubated with 1 μmol/L of OMC was significantly higher (2.695 ± 0.242 g, *p* < 0.05) than the tension observed in the control group (1.973 ± 0.252 g). Concerning histamine, the tension observed in rings contracted with Hist after blocking H_2_ with cimetidine, was significantly higher than the tension observed in rings contracted only by Hist, all with pre-incubation with OMC (*p* < 0.05 or *p* < 0.001). Regarding HUA-rings contracted by BHI, an agonist of H_1_ receptors, the tension observed was significantly higher in the HUA-rings pre-incubated with 50 μmol/L of OMC than in the control HUA-rings (*p* < 0.05).

Stable contractions of pre-incubated rings contracted with BHI were obtained after 15 min for all pre-incubated arteries (data not shown). Regarding the joint application of cimetidine + His, the results showed stable contractions only in rings from the HUA control and HUA pre-incubated with 10 μmol/L of OMC (Supplementary Figure S3).

**Figure 7:**
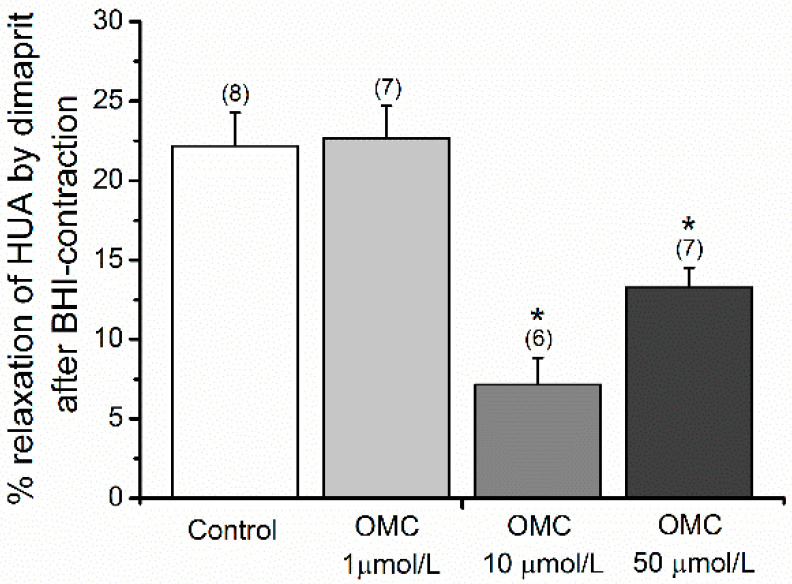
Percentage of relaxation induced by dimaprit (100 μmol/L), an H_2_ receptor agonist, on the human umbilical (HUA)-rings contracted with betahistine (BHI; H_1_ agonist; 1000 μmol/L) and incubated with octylmethoxycinnamate (OMC). Data are presented as mean percentage (bars) ± standard error of mean (S.E.M.) of the number *n* within brackets. * Represents a significant difference against HUA control (*p* < 0.05, one-way ANOVA with Dunnett’s).

The interference with H_2_ receptors, which induced relaxation when activated, was also assessed using dimaprit, receptor agonist. Figure 7 presents the results of dimaprit on HUA-rings contracted with the H_1_ receptor agonist (BHI). The percentage of relaxation was significantly lower (*p* < 0.05) in HUA-rings incubated with 10 μmol/L (7.148 ± 1.697%) and 50 μmol/L (13.265 ± 1.268%) OMC, when compared to HUA-rings from the control group (22.184 ± 2.080%).

### 3.6. Effects of OMC on the Expression of 5-HT and Hist Receptors

**Figure 8:**
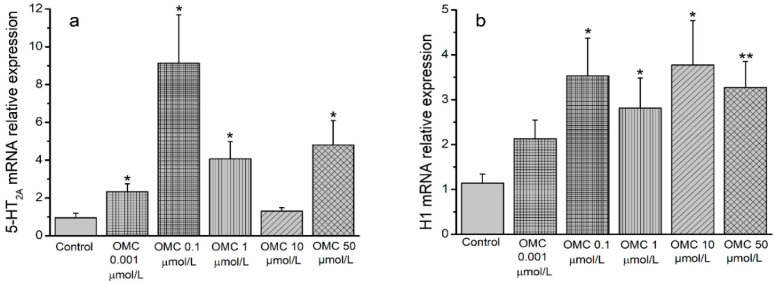
Relative expression of serotonin and histamine receptors in smooth muscle cells from the human umbilical artery (HUASMC) exposed to octylmethoxycinnamate (OMC; 0.001 μmol/L, 0.1–50 μmol/L): (**a**) expression of 5-HT_2A_ receptors and (**b**) expression of H_1_ receptors. Human β-actin was used as a housekeeping gene to normalize contractile agent receptor’s mRNA expression. Data are expressed as relative expressions. Bars represent the mean values and vertical lines represent the S.E.M of at least four different arteries. * Represents significant differences when compared to the control: * (*p* < 0.05) and ** (*p* < 0.01); Student’s *t*-test.

The effects of OMC on the expression of mRNA receptors involved in the contractile response to 5-HT (5-HT_2A_ receptors) and histamine (H_1_ receptors) are presented in Figure 8.

The results show a significantly higher mRNA relative expression of 5-HT_2A_ receptors in HUASMCs pre-incubated with all concentrations of OMC, except for the concentration of 10 μmol/L (Figure 8a). Similarly, an mRNA relative expression of H_1_-receptors was also significantly higher in HUASMCs pre-incubated with all OMC concentrations, except the lowest concentration, compared to the control group.

## 4. Discussion

The purpose of this work was to understand how the long-term exposures to the UV filter octylmethoxycinnamate (OMC), which is used daily by pregnant women, affects the vascular homeostasis of this sensitive group. The European Union determined that the final maximum concentration of OMC present in the active formulation of personal care products never exceeded 10% (*w/w*). As an ingredient of personal care products, the OMC is designed to cross skin layers, be metabolized, and then excreted. However, OMC can accumulate in some tissues [33] as the biotransformation process is not always fully effective. Indeed, Janjua, et al. (2008) [34] evaluated the topical application of 2 mg/cm^2^ of cream (which corresponds to 40 g for an average body area of 2.0 m^2^) in humans and detected OMC in plasma (0.016 μg/mL) and urine (0.006 μg/mL), corresponding to 0.055 μmol/L and 0.020 μmol/L, respectively. Thus, the range of concentrations used was from physiologic to pharmacologic (0.001–50 μmol/L).

Regarding the long-term effects of OMC in HUA contracted with 5-HT, exposure to increasing concentrations of OMC affected the artery vascular tone, inducing both vasorelaxation and vasocontraction depending on the pre-incubation concentrations. These results can be explained by the interaction between OMC pre-incubation and the different concentrations of OMC analysed. While lower concentrations of pre-incubation with OMC induced vasorelaxation of the HUA exposed to cumulative concentrations of OMC, intermediate OMC pre-incubation caused an opposite response, i.e., vasoconstriction. For the first time, these results suggest that OMC modulates vascular homeostasis by interference with serotonin receptors. Therefore, exposure to OMC might be harmful to the vascular system of pregnant women by inducing a pronounced vasorelaxation or vasoconstriction. These results are in accordance with several studies that associated prolonged exposure to EDCs with cardiovascular complications [16,35], including OMC [22].

Serotonin (5-HT) is the most potent vasoactive agent to contract HUA [36]. According to some authors, the contractile effects of this vasoactive agent are due to activation of different 5-HT receptors (5-HT_2A_, 5-HT_1B_/5-HT_1D_, and 5-HT_7_), which are present in HUA [17,37].

Our results suggest that OMC might interfere with these receptors as an interaction between the OMC incubation and the agonists of 5-HT receptors was observed. Indeed, the contractile response of HUA incubated with lower OMC concentrations, induced by the 5-HT_2A_ receptor agonist, was higher than in the HUA without OMC incubation. Moreover, the contractile response induced by this agonist was higher than that caused by 5-HT for the HUA incubated with an intermediate OMC concentration. These results suggest that OMC increases the activity of these receptors and that the contraction induced by 5-HT in the HUA incubated with OMC is mainly due to the activation of 5-HT_2A_ receptors, acting by the PLC/IP3 pathway, as reported in a previous study for non-incubated HUA [17].

The agonist of 5-HT_1B_ and 5-HT_1D_ receptors induced a smaller contractile response than that caused by 5-HT in non-incubated HUA; this agreed with other author’s findings [17]. However, the same does not apply for the HUA incubated with OMC. The contractile response in HUA incubated with lower OMC concentrations induced by an agonist of 5-HT_1B/1D_ receptors was similar to that induced by 5-HT; however, it was higher in HUA incubated with the highest OMC concentration. Moreover, OMC increased the contractile response induced by the 5-HT_1B_/_1D_ receptor agonist in incubated HUA. However, some arteries remained unresponsive to this agonist, which was also observed in previous studies on this artery [17,37]. The number of unresponsive HUAs was more pronounced for HUAs incubated with the higher concentrations of OMC. However, the initial contractile response to the agonist in these HUAs was more significant. This effect clearly shows a change in 5-HT_1B/1D_ receptors, since some receptors are not active; those that are active increase their activity to compensate the others. Therefore, taken together, these results suggest, despite some HUA variability, OMC affects the activity of 5-HT_1B_ and 5-HT_1D_ receptors, promoting the contraction of the artery through inhibition of adenyl cyclase.

Regarding the involvement of 5-HT_7_ receptors, the agonist of this receptor induced relaxation of the control arteries contracted with the 5-HT_2A_ receptor agonist, as expected. A previous study with HUA contracted with AMHT (5-HT_2A_ agonist) also observed an induction of relaxation with AS19, the agonist of the 5-HT_7_ receptor [17]. However, when incubated with OMC and contracted with AMHT, the HUA loses the ability to relax in the presence of the 5-HT_7_ receptor agonist. Regarding the response of arteries contracted with the agonist of 5-HT_1B_/_1D_ receptors (L69), significant variability was observed, since some control arteries relaxed and others contracted due to the 5-HT_7_ agonist, which led to a final effect close to zero. According to the effect of OMC incubation on the response to the 5-HT_7_ agonist, the same variability was observed. The HUA incubated with the intermediate concentration of OMC only constricted, while a higher incubation concentration induced relaxation. These results suggest that OMC can affect the capacity of the artery to relax through 5-HT_7_ receptors, probably blocking or inhibiting the receptor or adenyl cyclase activation.

According to the organ bath experiments, where OMC increased the activity of 5-HT_2A_ receptors, higher mRNA expression levels for the 5-HT_2A_ receptors were also observed in the HUA exposed to OMC. Therefore, further analysis of the mRNA expression levels of 5-HT_1B/1D_ receptors may be the next step to prove the effect of OMC on serotonin receptors.

Regarding the long-term effects of OMC on the vascular tone of the HUA contracted with His, since a sustained contraction was only attained for the control HUA, the HUAs were not further exposed to OMC. In this sense, to better understand how OMC disrupts vascular homeostasis by Hist receptors and, thus, induces non-sustained contractions, the long-term effects of this UV-B filter on vascular responses to agonists and antagonists of different Hist receptors were analysed. The results showed that contrary to Hist (unspecific agonist of Hist receptors), BHI (an agonist of H_1_ receptors) induced stable contractions. Furthermore, no interaction between the OMC incubation and the agonists/antagonists of Hist receptors was observed. Thus, the OMC incubation did not influence the effect of agonists/antagonists of Hist receptors. Furthermore, the agonist of H_1_ receptors did not alter the tension of the HUA concerning Hist, as has been reported by other investigations with non-incubated HUA [17]. Contrary, arteries incubated with the highest concentration of OMC induced a higher tension with the H_1_ receptor agonist when compared to the tension induced by Hist, evidencing that something happens on the H_1_ receptor at this incubation concentration.

To unravel if H_2_ receptors are involved in non-sustained contractions induced by histamine, firstly, HUA rings were exposed to cimetidine, a H_2_ receptor antagonist, to block them. OMC caused a higher tension at 1 μmol/L of OMC incubated HUA by joining cimetidine + His. Moreover, when H_2_ was blocked, a higher tension was produced in HUA by Hist, independent of the OMC incubation concentration. Next, sustained contractions of contracted HUA rings by joint application of cimetidine + Hist were obtained for the non-incubated HUA and HUA incubated with 10 μmol/L of OMC. According to the results of the activation of the H_2_ receptors, as expected, and in accordance with Santos-Silva et al. (2009) [17], the H_2_ receptors agonist induced relaxation of the HUA control. However, the higher incubation OMC concentrations induced lower relaxation, suggesting that OMC interferes with the H_2_ receptor.

In summary, the results concerning Hist-contracted HUA are suggestive that: (1) OMC greatly enhances H_1_ receptor activity in arteries incubated with lower concentrations of OMC. This effect is reflected in two ways: on the one hand, a higher tension was produced by cimetidine + Hist, than that produced by Hist alone; and on the other hand, there is a drastic increase in initial tension, which causes a contraction decay over time (non-sustained contraction). (2) The H_1_ activity was increasing in HUA incubated with an intermediate concentration of OMC, since the tension produced by His, in the presence of the H_2_ receptor antagonist, was higher. However, this increase seems to also be influenced by the H_2_ receptor. Since a significant decrease in relaxation was observed in arteries contracted by the H_1_ receptor agonist, and a sustained contraction was observed when cimetidine + His was applied, this indicates that OMC can decrease the vasorelaxation induced by the H_2_ receptor. Thus, differences in tension produced by Hist and by cimetidine + Hist may not only be due to an increase in the H_1_ receptor activity but also due to a blockage of the H_2_ receptor. (3) Highest concentrations of OMC decreases the relaxation of arteries contracted by the H_1_ receptor agonist, suggesting that OMC may influence the H_2_ receptor. However, a non-sustained contraction was observed with the joint application of cimetidine + His. The tensions were similar to those produced by BHI or Hist alone, also evidencing the interference of OMC with the H_1_ receptor. The higher mRNA expression levels for the H_1_ receptors observed in HUA exposed to OMC are in accordance with the results from the agonists/antagonists, evidencing that OMC increases the activity of H_1_ receptors. Further analysis of the H_2_ receptor’s mRNA expression levels will be of utmost interest to prove the OMC effect on H_2_ receptors.

Overall, our results concerning the long-term effects of OMC clearly demonstrated that OMC alters the vascular homeostasis of the HUA. As reported by other authors who studied the long-term effects of another EDC (tributyltin, TBT) on the HUA [30], here, the OMC also seems to increase 5-HT_1B/1D_ receptors. However, contrary to TBT, the activation of 5-HT_2A_ receptors seems to continue to be responsible for the contractions of HUA exposed to OMC. Thus, the effect of OMC appears to be due to the modulation of this UV filter, mainly on 5-HT_2A_ (activating the PLC/IP_3_ signalling cascade) and, also, on 5-HT_1B/1D_ (inhibiting adenyl cyclase activation) receptors. Moreover, OMC decreases/inhibits the activity of the 5-HT_7_ receptors in the HUA contracted with an agonist of 5-HT_2A,_ also suggesting the blocking or inhibiting of adenyl cyclase activation, and decreased vasorelaxation is observed. On the other hand, when a contraction is observed, the receptors seem to be “saturated.” This is suggestive of an effect of OMC that goes beyond a receptor’s interference. In this sense, further studies are needed to unravel the vascular MOA of OMC, which may also be related to the Ca^2+^ and/or K^+^ channels. The activation of ion channels could also explain the exaggerated relaxation observed in HUAs incubated with 1 μmol/L of OMC. Concerning the contractile responses in HUAs contracted with His, it was clearly demonstrated that OMC interferes with H_1_ receptors by activating the PLC/IP_3_ signalling cascade and with the H_2_ receptors by inhibiting the adenyl cyclase activation in a concentration-dependent manner. Gokina et al. (2000), in their work with rabbit cerebral arteries [38], demonstrated that the sustained contractions of Hist might not only be due to an increase in Ca^2+^ currents through VOCC and sensitization of the contractile apparatus, but also due to the non-selective cationic channels.

As evidence, the effects of long-term exposure to OMC denote clear interference with serotonin and histamine receptors. This interference with the receptors is extremely worrying for maternofetal health, as the HUA is devoid of innervation. Therefore, its physiological regulation is the responsibility of local mediators, such as serotonin and histamine [23]. Changes in the level of receptors of these mediators induce gestational hypertension and PE, thus compromising maternofetal health [17]. Indeed, HDP are a current public health problem. However, their treatment still generates much controversy and has not been fully clarified [39,40]. Investigating how certain substances are involved in these pathophysiological processes is crucial to contribute to their efficient treatment. Some studies have already suggested that serotonin receptors may be involved in contractile responses in these HDP [26,37,41,42,43,44]. Elevated serotonin levels present in the plasma and placenta of preeclamptic women evidenced the participation of serotonin in PE [24,25,26]. High histamine levels have also been associated with PE, which increases the HUA reactivity/sensitivity to these vasoactive agents, increasing vascular resistance [27,28].

Consequently, an increase in HUA contractile activity leads to a decrease in maternal–fetal blood flow, one of the main causes of intra-interim death. Therefore, to improve maternofetal health (which has remained so neglected over the past few years), physiological and pharmacological studies should be carried out to clarify the mechanisms involved in the contractility of this artery, and how certain daily used products, acting as EDCs, impair it. These studies will open new horizons in the treatment of HDP, such as PE and gestational hypertension.

From a clinical point of view, one of the biggest challenges is to understand the dose–response in and adjacent to the therapeutic zone. Indeed, it seems that the traditional accepted models, for the threshold and linear dose–response of the effects of different drugs, fail to give reliable estimates, particularly when lower concentrations are addressed. Indeed, non-monotonic relationships are a rather common effect for EDCs [45], where the existence of effects at lower and higher exposure levels, and diminished or non-existent toxicity at intermediate exposure levels, make it even more difficult to ascertain these disruptive mechanisms and define threshold limits. In a sense, the concept of hormetic dose Ensure meaning is retained response emerged with quite a favourable performance when it comes to chemical hazard/risk assessment in toxicological and pharmacological investigations, as is in the case of the present study. Hormesis is characterised as a dose–response phenomenon of low-dose stimulation and high-dose inhibition [46] and is generally accepted together with low-dose toxicity as a mechanism of endocrine disruption. While, in toxicology, the mechanisms of additivity and synergy are well established, studies are more limited concerning hormetic dose responses. Even so, it is accepted that the maximum responses in the hormetic stimulating zone are still restricted to a 30 to 60% increase in amplitude [47], which is in line with our results. Therefore, considering the disruptive responses induced by OMC in the present work, it seems acceptable to suggest that this UV filter has an EDC hormesis behaviour, which is particularly important, not only at a clinical level, but also in the evaluation of its environmental toxicity [47].

Recently, we reviewed that OMC leads to the formation of reactive oxygen species and has a potential to damage DNA [2], and therefore the use of antioxidants as UV filter stabilizers presented a novel promising strategy. Azizoglu et al. (2017) developed a combination therapy of OMC with the antioxidant, melatonin, which demonstrated permeation (7.40% and 58%) and accumulation after 24h (27.6% and 37%) for OMC and melatonin, respectively. The combination therapy allowed OMC to only accumulate in the upper skin while melatonin penetrated the deeper layers, highlighting what should be the ideal sun protection effect in the prevention of skin cancer after exposure to UV radiation [48].

Taking the concept of hormesis as a basis, we can infer that the preconditioning signal leading to cell protection is thus an important age-dependent redox, associated with ROS accumulation, and inflammatory responses involved in the pathophysiology of skin ageing [49]. This disruption of skin cell protection and stress survival is based on a relationship between the redox state and the vitagene network playing a key role, at the biological level, in the resilience mechanisms underlying the pathophysiology of oxidative stress [50]. In other words, the hormetic dose–response should be seen as a reliable feature of the dose–response to oxygen free radicals and their redox-regulated transcriptional factors, as well as antioxidant compounds. It appears to have an important impact on the pathophysiology of skin and the mechanisms of stress resilience to oxidative and inflammatory insults and degenerative damage induced by photoaging. Worryingly, ROS production can impair the fundamental functions of cell membranes (involving their receptors and signalling pathways), resulting in cell damage and death [46]. Therefore, knowledge of these signalling pathways, taking the concept of hormesis as a basis, is fundamental to the treatment of human diseases, such as CVD [46], which involve cellular damage by free radicals, that antioxidants neutralise.

On the other hand, the knowledge about sun exposure, cosmetic products use, and vitamin D status is a particularly important public health issue, especially for pregnant women. Sun protection present in cosmetic products is designed to prevent skin cancer, but recently some questions have been raised about the fact that it may induce a vitamin D deficit. In fact, this issue is even more relevant for pregnant women, because the literature is consistent, showing that vitamin D deficiency is common during pregnancy and may present some adverse outcomes for maternal and neonatal health (please see the review [51]). This issue raises some concerns about the use of cosmetic products in this sensitive window of development. According to the recent review by Neale et al. (2019), experimental studies suggest that sunscreens may block vitamin D synthesis in the skin; however, observational studies disagree with this theory, stating that real-life use of these products has a very low risk of interfering with its synthesis. Thus, the authors recommend that sun protection care should not be abandoned but highlight that sunscreen, which is widely recommended (where solar protection factor is sufficiently high) have not been studied so far, in relation to this issue [52]. On the other hand, some research has highlighted the protective role of the vitamin D endocrine system in HDP, such as PE, acting at a vascular level, through the regulation of SMC proliferation and angiogenesis, as well as through the reduction in cholesterol uptake by arterial cell walls [51]. Recently published studies, in a systematic review and meta-analysis [53], and in a cohort study [54], highlight the possibility of a strong association between lower vitamin D levels with the risk of developing PE. Concordantly, vitamin D supplementation during pregnancy has also been associated with a reduced incidence of PE [55,56] and improved with medical treatment with nifedipine in these pathological conditions [57]. At this moment, there is insufficient scientific evidence for vitamin D supplementation/treatment during pregnancy, but it presents itself as a potential appropriate gestational intervention strategy to prevent PE [51]. Further studies should be conducted to clarify the role of vitamin D in improving maternal–fetal health, namely by assessing the real influence of cosmetic products on the status of this vitamin and the possible implications during pregnancy.

## 5. Conclusions

Our results demonstrated that the long-term exposure of the UV filter OMC, used daily by pregnant women, affects vascular homeostasis. Furthermore, it also revealed that OMC modulates the 5-HT (5-HT2A, 5-HT1B/1D, and 5-HT7) receptors involved in the contractile process in a concentration-dependent manner. This modulation reflected a contraction or an exacerbated vasodilation, two effects harmful for the vascular system of pregnant women. Also, OMC clearly modulates Hist (H1 and H2) receptors, directly or indirectly, evidenced by non-sustained contraction. These findings suggest an increase in reactivity of the HUA to 5-HT and Hist as a result of OMC exposure. Worryingly, all these alterations can induce PE and/or gestational hypertension. In this sense, this study opens new horizons for the prevention and treatment of hypertensive diseases associated with pregnancy. Subsequent studies are needed to better clarify the vascular MOA of OMC due to a daily and, consequently, long-term exposure to this UV filter to establish a correlation of their effects with the incidence of cardiovascular disease in this sensitive population.

## Figures and Tables

**Table 1:** Primers Used for Real-Time PCR Experiments.

Gene	GenBank Accession No.	Primer (5′-3′)	Annealing Temperature (°C)
*β-actin*	NM_001101.5	Forward: 5-CAT CCT CAC CCT GAA GTA CCC-3 Reverse: 5-AGC CTG GAT AGC AAC GTA CAT G -3	60
*5-HT_2A_*	NM_001165947.5	Forward: 5-TCT TTC AGC TTC CTC CCT CA-3 Reverse: 5-TGC AGG ACT CTT TGC AGA TG -3	60
*H_1_*	NM_001098212.2	Forward: 5-CAC ACT GAA CCC CCT CAT CT-3 Reverse: 5-GGC CTT CGT CCT CTA TTT CC-3	60

## Data Availability

The data presented in this study are available within the article or supplementary material.

## Supplementary Materials

The following supporting information can be downloaded at: https://www.mdpi.com/article/10.3390/biomedicines10051054/s1, **Table S1:** Basal tension of the human umbilical artery (HUA)-rings exposed to octylmethoxycinnamate (OMC, 0.001–50 µmol/L) after an incubation with OMC (0, 1, 10 and 50 µmol/L). Values are presented as mean of the basal tension ± S.E.M.; **Table S2**: Tension in human umbilical artery (HUA)-rings incubated with octylmethoxycinnamate (OMC) contracted by the L69 (5-HT_1B/D_ agonist; 1 μmol/L). The contractile dual effect is represented by responsive and unresponsive HUA-rings. Each value represents the mean of the tensions ± S.E.M. and the *n* is the number within brackets. * Represents significant differences between responsive and unresponsive HUA-rings (*p* < 0.05, Student’s t-test); **Figure S1:** Percentage of relaxation of human umbilical artery (HUA)-rings incubated with (**A**) 0 µmol/L, (**B**) 1 µmol/L, (**C**) 10 µmol/L and (**D**) 50 µmol/L of octylmethoxycinnamate (OMC), contracted with serotonin (5-HT; 1 µmol/L) and exposed to cumulative concentrations of OMC (0.001–50 μmol/L). Each point represents the mean and vertical lines represent the S.E.M. * Represents a significant difference when compared with the respective solvent control: * (*p* < 0.05), ** (*p* < 0.01), *** (*p* < 0.001); and the different letters represents significant differences between OMC concentrations (*p* < 0.05); two-way ANOVA method followed by Holm–Sidak post hoc tests; **Figure S2:** Real tension induced by histamine (Hist; 10 μmol/L) on human umbilical artery (HUA)-rings incubated with different octylmethoxycinnamate (OMC) concentrations (0, 1, 10, and 50 μmol/L). Data are expressed as real tension in grams (g) of at least three different arteries; **Figure S3:** Real tension induced by histamine (Hist; 10 μmol/L) after adding cimetidine (Cim, H_2_ antagonist, 10 μmol/L) on human umbilical artery (HUA)-rings incubated with different octylmethoxycinnamate (OMC) concentrations (0, 1, 10, and 50 μmol/L). Data are expressed as real tension in grams (g) of at least three different arteries.
