# Supplementary material for: UV-B Filter Octylmethoxycinnamate Is a Modulator of the Serotonin and Histamine Receptors in Human Umbilical Arteries
Source: Biomedicines. 2022 May 3;10(5):1054. doi: 10.3390/biomedicines10051054 (PMC9139053; doi:10.3390/biomedicines10051054)
Supplement: Supplementary file 1 [file biomedicines-10-01054-s001.zip › biomedicines-1714924-supplementary.pdf]

UV-B filter octylmethoxycinnamate is a modulator of the serotonin and histamine receptors in human umbilical arteries

Margarida Lorigo[1,2], Carla Quintaneiro[3], Luiza Breitenfeld[1,2] and Elisa Cairrao[1,2►]

Margarida Lorigo[1,2] (margarida.lorigo@gmail.com; ORCID ID: 0000-0002-4752-6398), Carla Quintaneiro[3] (cquintaneiro@ua.pt; ORCID ID: 0000-0002-1151-8177), Luiza Breitenfeld[1,2] (luiza@fcsaude.ubi.pt; ORCID ID: 0000-0001-8550-1645) and Elisa Cairrao[1,2►] (ecairrao@fcsaude.ubi.pt; ORCID ID: 0000-0002-4823-5701)

[1] CICS-UBI, Health Sciences Research Centre, University of Beira Interior, 6200-506, Covilhã, Portugal.

[2] FCS - UBI, Faculty of Health Sciences, University of Beira Interior, 6200-506 Covilhã, Portugal

[3] Department of Biology & CESAM (Centre for Environmental and Marine Studies), University of Aveiro, 3810-193 Aveiro, Portugal

► **Corresponding author:**

Elisa Cairrao, PhD

Health Sciences Research Centre

University of Beira Interior

Av. Infante D. Henrique

6200-506 Covilhã

Portugal

Tel.: +351-275-329049

Fax: +351-275-329099

e-mail: ecairrao@fcsaude.ubi.pt

ORCID iD: https://orcid.org/0000-0002-4823-5701

Supplementary Material

Table S1 Basal tension of the human umbilical artery (HUA)-rings exposed to octylmethoxycinnamate (OMC, 0.001–50 µmol/L) after an incubation with OMC (0, 1, 10 and 50 µmol/L). Values are presented as mean of the basal tension ± S.E.M

	Basal Tension of OMC (mN)			
**OMC (µmol/L)**	Without incubation (n=7)	Incubation of 1 µmol/L (n=12)	Incubation of 10 µmol/L (n=7)	Incubation of 50 µmol/L (n=12)
0.001	-0.40 ± 0.29	0.17 ± 0.16	0.13 ± 0.16	0.11 ± 0.27
0.01	-0.17 ± 0.27	-0.10 ± 0.21	0.17 ± 0.26	-0.42 ± 0.17
0.1	-0.22 ± 0.25	-0.19 ± 0.28	-0.03 ± 0.36	-0.68 ± 0.19
1	-0.36 ± 0.33	-0.13 ± 0.32	0.20 ± 0.38	-1.33 ± 0.32
10	-0.32 ± 0.29	-0.28 ± 0.37	-0.08 ± 0.50	-1.26 ± 0.29
50	-0.28 ± 0.34	-0.02 ± 0.43	-0.16 ± 0.62	-1.42 ± 0.34

Table S2 Tension in human umbilical artery (HUA)-rings incubated with octylmethoxycinnamate (OMC) contracted by the L69 (5-$HT_{1B/D}$ agonist; 1 µmol/L). The contractile dual effect is represented by responsive and unresponsive HUA-rings. Each value represents the mean of the tensions ± S.E.M. and the n is the number within brackets. * Represents significant differences between responsive and unresponsive HUA-rings ($p<0.05$, Student's t-test)

	Tension of HUA-rings contracted by L69 (mN)	
**OMC (µmol/L)**	Responsive HUA-rings	Unresponsive HUA-rings
0	9.74 ± 0.89 (n=7)	3.39 ± 0.97 (n=9) *
1	17.71 ± 1.58 (n=6)	2.88 ± 1.06 (n=8) *
10	18.56 ± 2.52 (n=8)	3.05 ± 0.68 (n=17) *
50	27.12 ± 4.64 (n=7)	2.94 ± 0.37 (n=26) *

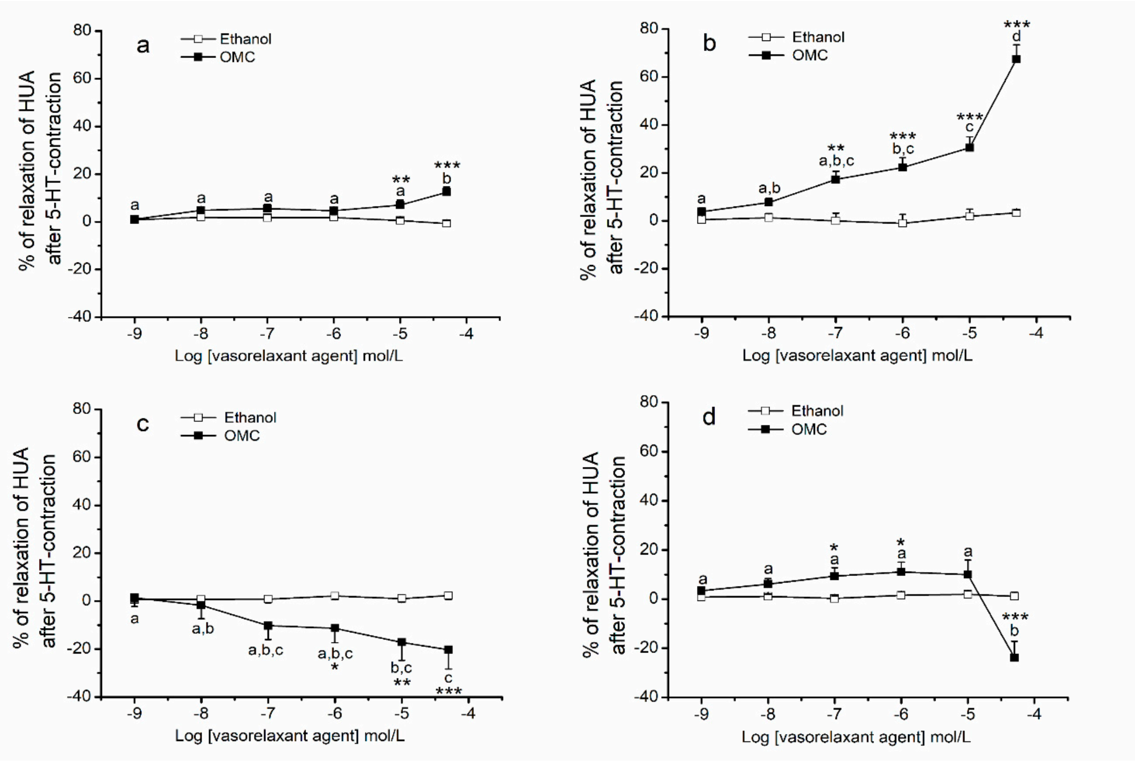

Figure S1 Percentage of relaxation of human umbilical artery (HUA)-rings incubated with **(A)** 0 µmol/L, **(B)** 1 µmol/L, **(C)** 10 µmol/L and **(D)** 50 µmol/L of octylmethoxycinnamate (OMC), contracted with serotonin (5-HT; 1 µmol/L) and exposed to cumulative concentrations of OMC (0.001 µmol/L-50 µmol/L). Each point represents the mean and vertical lines the S.E.M. * Represents a significant difference when compared with the respective solvent control: * ($p<0.05$), ** ($p<0.01$), *** ($p<0.001$); and the different letters represents significant differences between OMC concentrations ($p<0.05$); two-way ANOVA method followed by Holm-Sidak post-hoc tests

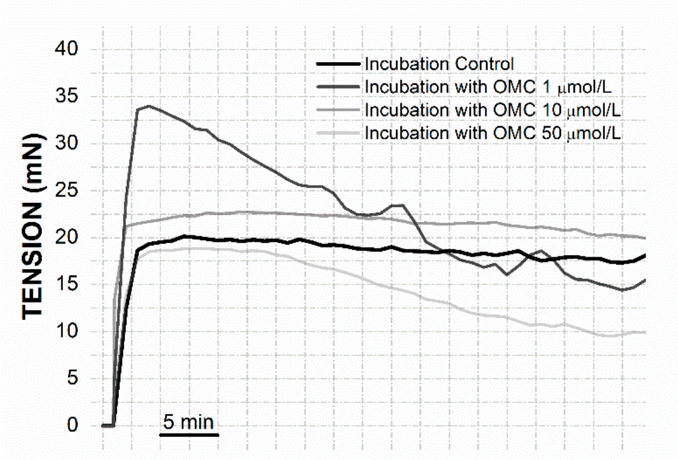

Figure S2 Real tension induced by Histamine (Hist; 10 µmol/L) on human umbilical artery (HUA)-rings incubated with different octylmethoxycinnamate (OMC)-concentrations (0, 1, 10 and 50 µmol/L). Data are expressed as real tension in grams (g) of at least three different arteries

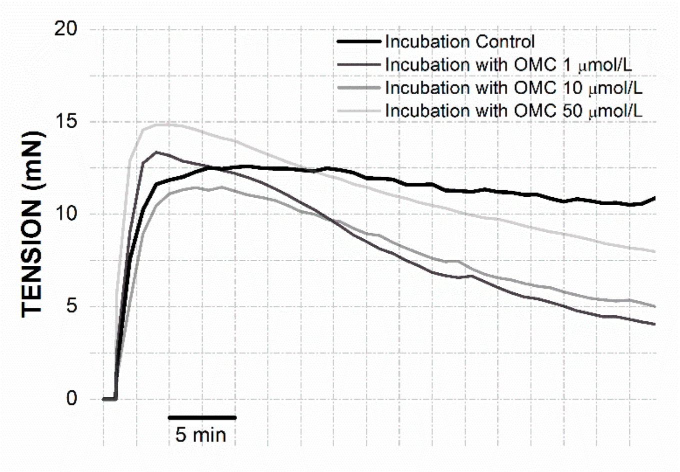

FigureS3 Real tension induced by Histamine (Hist; 10 μmol/L) after adding cimetidine (Cim, H_2 antagonist, 10μmol/L) on human umbilical artery (HUA)-rings incubated with different octylmethoxycinnamate (OMC)-concentrations (0, 1, 10 and 50 μmol/L). Data are expressed as real tension in grams (g) of at least three different arteries
